# Mortality and diurnal temperature range in Virginia

**DOI:** 10.1007/s00484-025-02850-6

**Published:** 2025-01-30

**Authors:** Robert E. Davis, Owen Himmel, Parker K. Sims, Christopher M. Fuhrmann

**Affiliations:** 1 Department of Environmental Sciences, University of Virginia, P.O. Box 400123, Charlottesville, VA 22904-4123 USA; 2 Southeast Regional Climate Center, Department of Geography and Environment, University of North Carolina, Chapel Hill, NC 27514 USA

**Keywords:** Diurnal temperature range, Temperature variability, Mortality, Virginia, Distributed lag non-linear model, Spatial synoptic climatology

## Abstract

The relationship between diurnal temperature range (DTR) and daily mortality from 2005 to 2020 is examined for seven large metropolitan areas in Virginia using distributed lag non-linear models that control for temperature and humidity. The relative risk of mortality increases for very high DTR, and there is a short lag effect of several days. High risk DTR days are rare, typically occurring less than 1% of the time at most locations. These days primarily occur in spring and are characterized by high pressure and low humidity that allow overnight temperatures to drop substantially. High DTR days are often associated with elevated respiratory and cardiovascular mortality and tend to impact the elderly. The similarity between the mortality response to high DTR and that of high temperatures alone, coupled with the lack of clear physiological underpinnings, challenges the notion that these responses are independent. The prevalent spring peak in dangerous DTR days suggests that lack of acclimatization to hot and humid conditions may impose strain on the cardiovascular and respiratory systems.

**Supplementary Information:**

The online version contains supplementary material available at 10.1007/s00484-025-02850-6.

## Introduction

There is widespread and growing evidence of a connection between diurnal temperature range (DTR, the difference between the daily maximum and minimum temperature) and human health, with poor outcomes associated with days having a large DTR (Cheng et al. 2014). In most of these studies, some effort is made to (statistically) control for temperature (e.g., Kan et al. 2007; Lim et al. 2012; Zhou et al. 2014; Ding et al. 2015). Thus, the implication is that short-term temperature *variability* alone exerts enough physiological strain on the body to enhance human morbidity.

A nuanced understanding of how and why DTR impacts human health is currently lacking. For example, one might logically hypothesize that low nighttime temperatures during a heat wave would be preferable to warm nights, just as warm days would be beneficial during a cold snap. But statistical analyses seem to indicate otherwise, if, in fact, the DTR effects are independent of temperature. We similarly lack a clear understanding of the specific weather conditions on these detrimental DTR days. This is especially important if we are to identify the underlying factors that might be driving this physiological response.

In this analysis, we leverage a high-quality, patient-level, long-term mortality dataset for the Commonwealth of Virginia to explore specifically the relationships between DTR and mortality. We first develop models for areas in and around seven large metropolitan areas of Virginia to examine the nature of the DTR-mortality relationship across the state. We then examine, in detail, the climate of high DTR days to provide a stronger understanding of the weather conditions and seasons associated with the highest DTR mortality. We conclude by exploring the diseases most strongly associated with mortality on these extreme DTR days and the demographic characteristics of the most affected groups. Thus, we hope to shed light on DTR-health associations by explicitly examining patient-level health outcomes through the lens of high DTR events.

## Background

Numerous studies have identified relationships between DTR and mortality in varied locations globally (Kan et al. 2007; Lim et al. 2012, 2015; Cheng et al. 2014; Zhou et al. 2014; Ding et al. 2015, 2016; Kim et al. 2016; Lee et al. 2018a; 2018b, 2019; Tang et al. 2018; Sharafkhani et al. 2019; Kai et al. 2023; Amoatey et al. 2024). In some cases, the relationships were found to be positive and linear, with a small increase in mortality for each degree increase in DTR (e.g., Kan et al. 2007; Lim et al. 2012; Kim et al. 2016; Amoatey et al. 2024) whereas other studies uncovered more U- or J-shaped relationship, with mortality increasing as some high DTR value is approached or exceeded (e.g., Ding et al. 2015; Kai et al. 2023).

The diseases most frequently linked to high DTR are respiratory (Kan et al. 2007; Cheng et al. 2014; Zhou et al. 2014; Lim et al. 2015; Ding et al. 2016; Kim et al. 2016; Tang et al. 2018) and circulatory/cardiovascular conditions (Kan et al. 2007; Song et al. 2008; Cheng et al. 2014; Zhou et al. 2014; Lim et al. 2015; Ding et al. 2016; Kim et al. 2016; Lee et al. 2018b; Tang et al. 2018). Elevated mortality risk to the elderly is particularly common (Cheng et al. 2014; Zhou et al. 2014; Lim et al. 2012, 2015; Ding et al. 2016; Kim et al. 2016; Tang et al. 2018; Kai et al. 2023; Amaotey et al. 2024), whereas a few studies found relationships to younger individuals (Cheng et al. 2014; Ding et al. 2015; Tang et al. 2018). Likewise, there is some inconsistency regarding whether DTR primarily impacts males (Ding et al. 2015; Tang et al. 2018; Kai et al. 2023) or females (Lim et al. 2012; Zhou et al. 2014; Tang et al. 2018).

The physiological connection that specifically links temperature *variability* to mortality or illness is lacking, although some studies have produced related results that might be relevant. Exposure to a short-term temperature increase could be related to coronary and cerebral thrombosis via impacts on heart rate, blood pressure and viscosity, red blood cell and platelet counts, and plasma cholesterol (Keatinge et al. 1986). There are some associations between daytime and nighttime blood pressure (Brook et al. 2011) that may be related to sleep quality, which itself is impacted by short-term temperature changes (Buquet 2007). However, there is some evidence that warm nights negatively impact sleep (Buquet 2007), a result that would imply an inverse relationship with DTR. Short-term cold exposure has been associated with inflammation and an increased likelihood for clot formation (Mercer et al. 1999). Inflammation in the nasal cavity has likewise been correlated to rapid temperature changes (Graudenz et al. 2006). Although quite a few studies associate temperature and humidity with physiological responses, almost none specifically examine temperature *change* as the independent driving factor.

Another important aspect of the connection between temperature variability and human health is the meteorological factors that influence seasonal and geographic patterns in DTR. There is ample evidence that DTR has decreased since the mid-20th century, primarily driven by rising minimum temperatures (Karl et al. 1993; Easterling et al. 1997; Braganza et al. 2004; Vose et al. 2005; Qu et al. 2014; Powell and Keim 2015; Sun et al. 2019). Some studies have also attempted to determine the causes of DTR variations and trends, which mostly relate to land use. Agricultural and forested lands tend to have low DTR because of higher soil moisture and evapotranspiration, which result in higher humidity and minimum temperatures (Bonan 2001; Jackson and Forster 2010; Scheitlin and Dixon 2010). Low DTR has also been observed over urban areas (Gallo et al. 1996; Scheitlin and Dixon 2010) and near bodies of water (Geerts 2003). Seasonally, low DTR has been found in winter and summer, while high DTR is more common in spring and autumn. These patterns have been tied to seasonal variations in humidity, cloud cover, and air mass type (Leathers et al. 1998; Durre and Wallace 2001; Geerts 2003; Sun et al. 2006; Scheitlin and Dixon 2010; Qu et al. 2014; Sun et al. 2019; Davis et al. 2020).

## Materials and methods

Daily mortality data were acquired from the Interjurisdictional Exchange Mortality File (death certificate records) from the Virginia Department of Health, Office of Information Management, for a 16-year period from 2005 to 2020. Each record contains the date of death, residence of the decedent, the primary and underlying cause(s) of death, and demographic information. Residence information was made available at the level of county or independent city. This research was determined to be “non-human subject research” by the Institutional Review Boards of the University of Virginia and the Virginia Department of Health.

Daily mortality totals were computed from the patient-level data for total mortality, as well as by eight age categories (≤ 11, 12–17, 18–29, 30–39, 40–49, 50–64, 65–74, and ≥ 75), by race (Black, White) and gender (Female, Male), based on the classification criteria utilized in the data files. Specific data on other categories (e.g., non-white Hispanic, Asian/Pacific Islander, race or gender unknown) were so incomplete as to make further analyses untenable for those groups. We also examined the primary cause of death according to the Major Diagnostic Category (MDC). The MDC combines the more specific diagnosis-related group into a broader classification that is largely based on organ systems. Thus, each mortality is classified into one and only one MDC, such as circulatory, respiratory, digestive, etc. A list of the MDCs is provided in Supplemental Table 1.

**Fig. 1:**
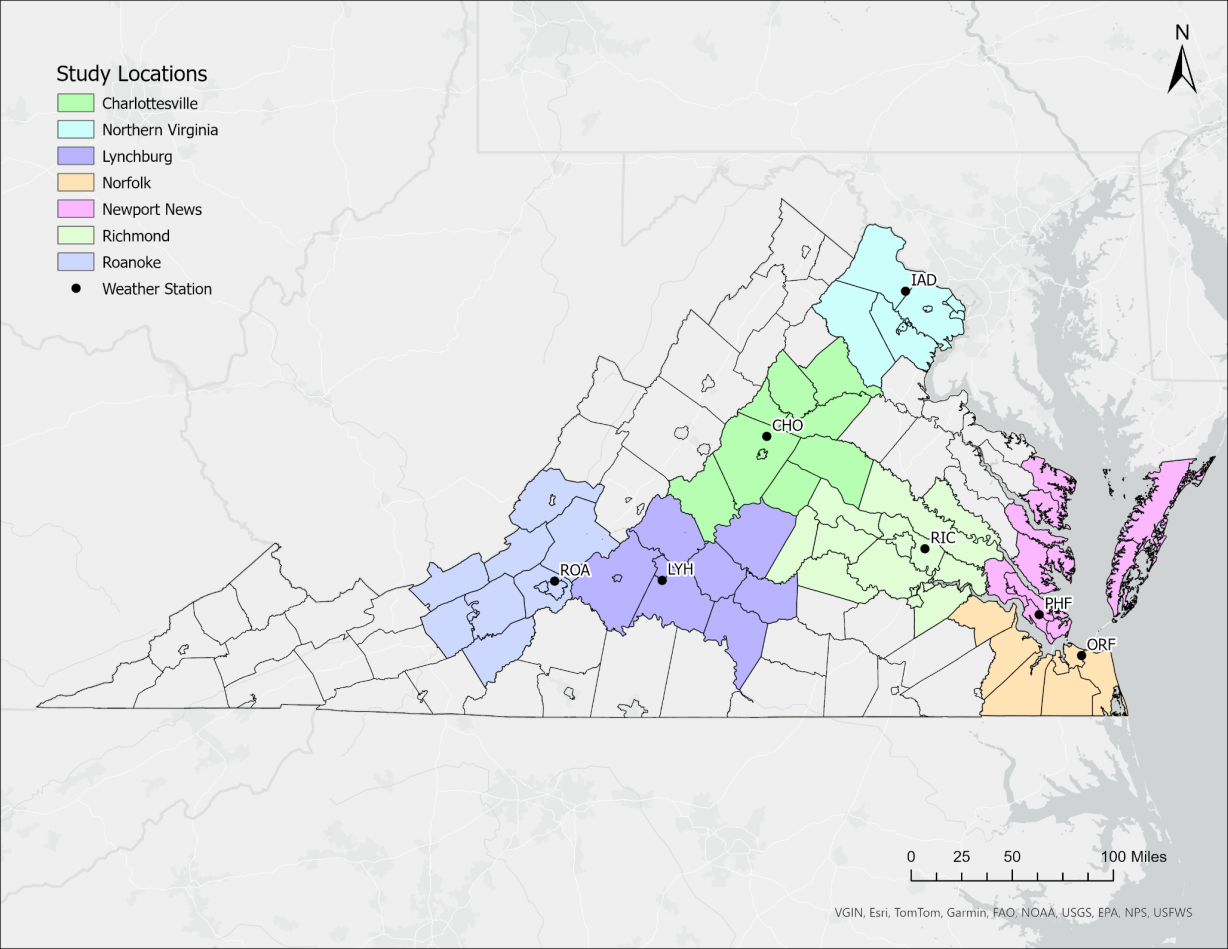
Locations of the Virginia counties associated with each of the seven study regions and the airport weather station associated with each region

Data were stratified by the residence of the decedent for seven major metropolitan areas in Virginia based upon the county or independent city (Fig. 1). The spatial footprint of each region was restricted to a local multi-county area to increase the likelihood that residents in each area experienced comparable weather conditions while factoring in local climate and topography (Davis et al. 2023). Climatological data for each region were associated with a single first-order weather station (Fig. 1). The locations examined were Northern Virginia (suburbs of Washington, D.C., weather station IAD, Dulles International Airport), Charlottesville (CHO, Charlottesville-Albemarle County Airport), Lynchburg (LYH, Lynchburg Regional Airport), Richmond (RIC, Richmond International Airport), and Roanoke (ROA, Roanoke-Blacksburg Regional Airport). The extensive metropolitan area of eastern Virginia was divided into northern and southern portions (Newport News (PHF, Newport News/Williamsburg International Airport) and Norfolk (ORF, Norfolk International Airport), respectively). A list of the counties and independent cities associated with each region, along with related sample sizes, can be found in Supplemental Table 2.

Meteorological observations were acquired for each weather station from the Automated Surface Observing Systems (ASOS) data archives, as overseen by the National Weather Service. Data were downloaded from the Iowa Environmental Mesonet website (https://mesonet.agron.iastate.edu/request/download.phtmlnetwork=VA_ASOS). For consistency, we acquired the observation closest to 0100, 0700, 1300, and 1900 Local Standard Time (LST). Any observation within 90 min of each time was included; if no observations were available across the 180-minute time window, that observation was coded as missing. A single missing observation was filled via linear interpolation, but two or more consecutive missing observations were not interpolated and were deemed to be missing. Weather variables downloaded were air temperature, dew point temperature, wind speed, and sea-level pressure.

Daily maximum and minimum temperatures were retrieved from the Global Historical Climate Network, which is managed by the National Centers for Environmental Information. Diurnal temperature range (DTR) was calculated as the difference between each day’s maximum and minimum temperature. The weather stations in this study record maximum and minimum temperature over the 24-hour period from midnight to midnight, so there is no concern that observation time would bias the results, as might occur at stations that only take morning measurements from a max/min thermometer.

In addition to the variables that were measured directly, a few additional parameters that are often used in climate-health research were computed. Apparent temperature (AT), which serves as the basis of the Heat Index used by the National Weather Service, was calculated using the Anderson et al. (2013) algorithm. Humidex (Hx) (Masterton and Richardson, 1981), a measure frequently used by the Canadian Weather Service, was calculated according to Davis et al. (2016). The Temperature-Humidity Index (a.k.a. “Discomfort Index” (Thom 1959), like AT and Hx, combines temperature and humidity but employs the wet bulb temperature, which was estimated from the air and dew point temperatures using the Normand (1946) approximation (Davis et al. 2016). Finally, the Wind Chill Index (Osczevski and Bluestein 2005) was estimated from the air temperature and wind velocity. Each of these parameters was calculated at 0100, 0700, 1300, and 1900 LST. The full list of variables can be found in Supplemental Table 3.

Furthermore, the Spatial Synoptic Classification version 3.0 (SSC) daily classification of weather types was examined as an additional climate parameter. The SSC uses multiple hourly observations of air temperature, dew point temperature, wind, pressure, and cloud cover to nominally classify each day into one of six weather types. Resulting types are classified based on humidity (dry (D) or moist (M)) and temperature (polar (P), moderate (M), or tropical (T)). A transition (TR) category is used for days with significant weather changes over 24 h. The SSC is a relative, seasonally standardized classification such that polar types may occur in summer and tropical classes are observed in winter (Sheridan 2002; https://sheridan.geog.kent.edu/ssc3.html).

Air quality data were downloaded from the Environmental Protection Agency archives (https://www.epa.gov/outdoor-air-quality-data/download-daily-data). Specifically, we acquired daytime (8 h) maximum concentrations of ozone and daily mean concentrations of particulate matter with a diameter ≤ 2.5 microns (PM_2.5_). In those locations with more than one air quality monitor, we converted each observation to a z-score using the mean and standard deviation of the observations for that monitor, then averaged z-scores across monitors to develop a regional mean value. Because the modeling approach is location-specific, we are not concerned with comparing air quality concentrations between sites, so this method retains the important local daily variability in air quality that is most relevant to this research.

For each city, distributed lag non-linear models (DLNMs) were used to relate the dependent variable (daily mortality count) to various potential predictor variables (Gasparrini, 2011). DLNMs are regression-based models that fit splines through predictor variables, which include a time component that accounts for long-term trends and seasonality. DLNMs employ a cross-basis function that combines the exposure response of a predictor with the associated lag response. Additional nominal and continuous variables can be added to the model to control for various confounders, similar to the approach of fitting multiple regression models. Because the dependent variable is daily mortality count, a quasi-Poisson link function is used to relate the potential predictors to mortality.

The general form of the model is:

1 $$\eqalign{ Log{\rm{ }}E{\rm{ }}\left( {{Y_t}} \right)\, = \, & intercept{\rm{ }} + {\rm{ }}cb{\rm{ }}\left( {DTR} \right){\rm{ }} + {\rm{ }}{s_1}\left( {time,{\rm{ }}16{\rm{ }}*{\rm{ }}{x_1}} \right) \cr & + {\rm{ }}{s_2}\left( {weather,{\rm{ }}{x_2}} \right){\rm{ }} + {\rm{ }}controls{\rm{ }} + {\rm{ }}factors \cr} $$

where Y is the daily mortality count on day t, cb is the cross-basis of DTR, s_1_ is a natural cubic-spline fit using x_1_ degrees of freedom for each of the 16 years, and s_2_ is a natural cubic spline fit through one or more weather variables with x_2_ degrees of freedom. “Controls” and “factors” account for continuous and nominal variables, respectively, that could be included in the model to incorporate various climatic and non-climatic factors that might influence the daily mortality count (Supplemental Table 3).

Specifically, the exposure-response of DTR was modelled using equally-spaced knots determined with a first-degree piecewise polynomial B-spline with 4 degrees of freedom. The lag response was modeled for a 21-day period using a natural cubic spline with 2 degrees of freedom, with equally-spaced knots on the log scale. A 21-day period was selected based on model testing and prior research (Ding et al. 2015; Kai et al. 2023), with the goal of selecting a long enough lag that exceeds the limit to which exposure to high DTR values might reasonably be expected to elicit a mortality response. Tests using a longer lag (28 days) showed that the model was largely unaffected for the shorter lags that are of primary interest to this study.

Various models were fitted and tested by adjusting the degrees of freedom in the spline terms and the variables included in the model. Models were compared using the quasi-Akaike Information Criterion (qAIC) and the residual deviance. All models consisted of a spline through the trend term, a DTR cross-basis, and at least one spline for a weather variable to control for temperature and/or humidity. This latter control is important as we wish to examine the impact of DTR that is independent of temperature. Variance inflation factors were used to examine the correlation between potential predictors.

For example, a typical model might be:

2 $$\eqalign{ Log{\rm{ }}\left( {mort} \right)\, = \, & cb\,\left( {DTR} \right)\, + \,ns\left( {time,{\rm{ }}16*3} \right)\, + \,ns\left( {Hx7am,{\rm{ }}3} \right)\, \cr & + \,SLP1pm\, + \,factor\left( {dow} \right) \cr} $$

where “mort” is the daily mortality, each of the 16 years in the “time” term is fitted with a natural cubic spline with 3 degrees of freedom, a spline with 3 degrees of freedom is fitted to 7 a.m. humidex, and 1 p.m. sea-level pressure (SLP) and day of week (dow) are included as controls.

Each of the 7 study cities was modeled separately. The output of the model is the relative risk (RR) of mortality as a function of DTR and lag. Based on these results, high RR values (DTR and lag combinations) in each city were isolated for more detailed analysis.

As a test of model robustness, each city was fitted with an identical model, with variables selected based on the consensus of the individual city models. The consensus model was:

3 $$\eqalign{ Log\left( {mort} \right)\, = & \,cb\,\left( {DTR} \right)\, + \,ns\,\left( {time,{\rm{ }}16*5} \right)\, + \,ns\left( {Hx1pm,{\rm{ }}3} \right)\, \cr & + \,RH7am\, + \,SLP1am\, + \,factor\left( {dow} \right) \cr} $$

where “RH7am” is 7 a.m. relative humidity. For details on the DLNM methods in general, please refer to Gasparrini (2011).

Mortality for various demographic and MDC categories was compared for high DTR days vs. all other days using independent samples t-tests (one-tailed) based on 1000 bootstrapped samples. P-values were adjusted based on Levene’s test for equality of variances. Finally, chi-square tests were used to compare SSC frequencies for days with high DTR vs. all other days. All tests were run with a type I error rate of 0.05. DLNM models were run using the “mgcv” package in R Studio, Version 2024.04.2 + 764.

## Results

The final DLNM model for each city included at least one temperature or humidity variable as a control (Table 1). Humidex was selected as the optimal variable in five of the seven cities, and THI was chosen in one. Both indices include thermal and humidity components. The temperature and humidity variables, except relative humidity, are highly correlated (Supplemental Table 4), so the impact of specific variable selection on the result is minimal. At PHF, morning dew point temperature was selected, but in this humid, coastal location, air and dew point temperature are very highly correlated (*R* = 0.996 at 7 a.m.), so this variable is effectively comparable to the other parameters that combine temperature and humidity explicitly. Depending on the location, controls were included for relative humidity, sea-level pressure, and PM_2.5_. Likewise, nominal factors for day of week and holidays (Thanksgiving and Christmas) were included in some cases.

**Table 1:** Summary of the models selected for each location (Fig. 1). The cross-basis is DTR. Each model includes splines for time and for a thermal/moisture variable. Additional splines, controls (continuous variables) and factors (nominal variables) may be added to improve model fit, based on the Quasi-Akaike’s Information Criterion (QAIC) and residual deviance (RESID. DEV.). Similar information is provided for a consensus model (CON). Df = degrees of freedom, Hx = humidex, SLP = sea-level pressure, RH = relative humidity, T_d_=dew point temperature, THI = temperature-humidity index, dow = day of week

	TIME (yr*df)	SPLINE (df)	CONTROLS	FACTORS	QAIC	RESID. DEV.	CON. QAIC	CON. RESID. DEV.
CHO	16*3	Hx7pm (3)	RH1pm	dow	25,845	5951	25,898	5946
		SLP7am (4)						
IAD	16*5	Hx1pm (2)	SLP1pm	dow	36,024	6011	36,059	6045
				holidays				
LYH	16*5	Hx1pm (2)	SLP1am		28,239	5784	28,248	5779
			RH7am					
ORF	16*5	Hx7am (3)	SLP7am	holidays	34,781	5835	34,838	5841
			RH7am					
			PM_2.5_					
PHF	16*3	T_d_7am (2)	RH1pm		31,842	5888	31,891	5848
		SLP7pm (3)	PM_2.5_					
RIC	16*3	Hx7am (3)	SLP1pm	dow	35,789	6055	35,866	6007
ROA	16*5	THI1pm (2)	RH7am		31,169	5678	31,206	5673
			SLP1pm					
CON	16*5	Hx1pm (3)	RH7am	dow				
			SLP1am					

**Fig. 2:**
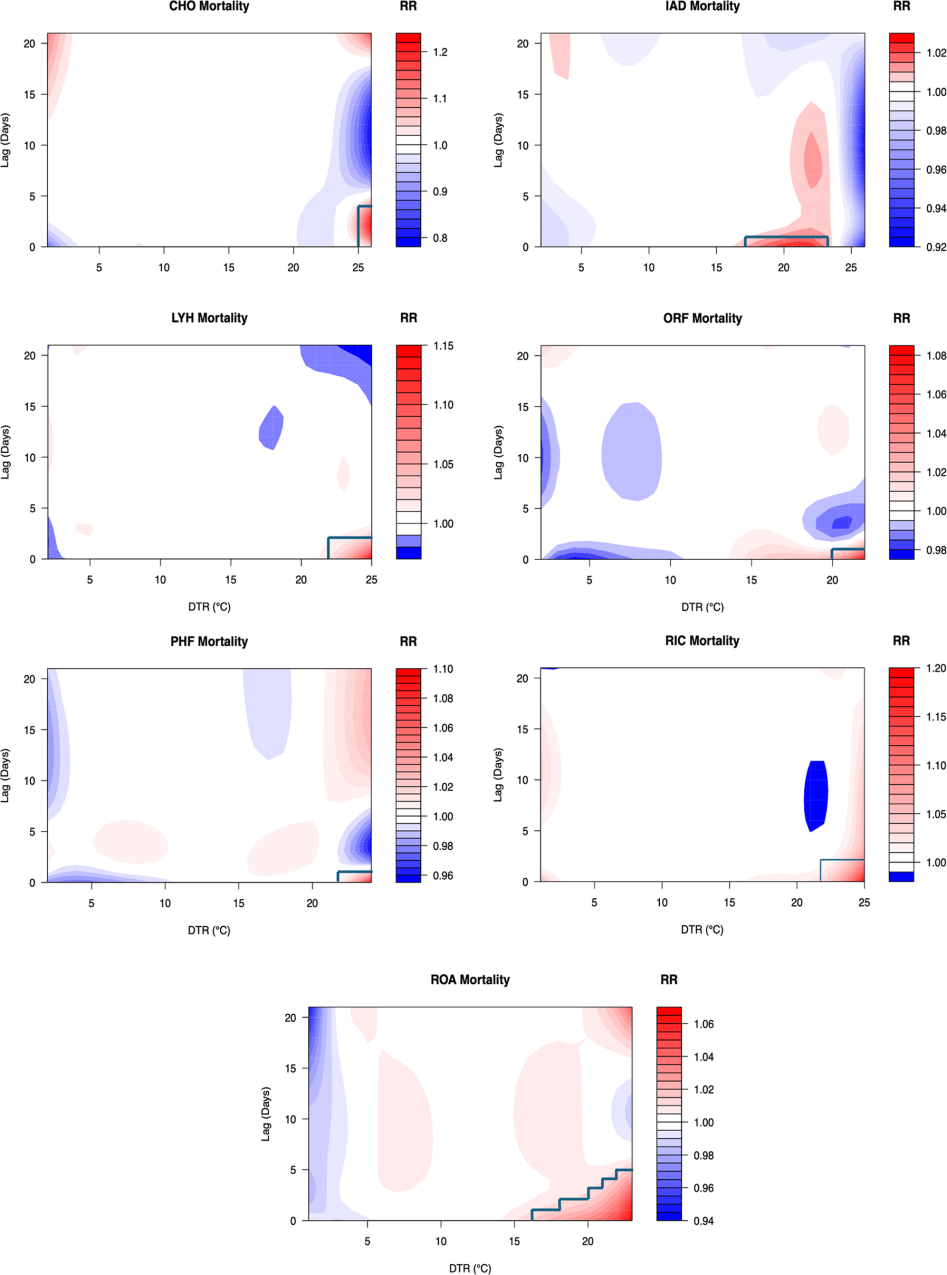
Relative risk of mortality as a function of diurnal temperature range (°C, x-axis) and lag (days, y-axis) at each location (IAD = Northern Virginia; CHO = Charlottesville; LYH = Lynchburg; PHF = Newport News; ORF = Norfolk; RIC = Richmond; ROA = Roanoke). High DTR days associated with elevated mortality are indicated by the green line (see Table 2). Note that the RR color scale varies between the panels

All seven cities exhibited increased RR for high DTR (Fig. 2). The maximum RR varied from 1.02 (IAD) to 1.20 (CHO and RIC). In general, the RR of mortality tended to increase with increasing DTR once a threshold was exceeded, and the lag effects were short (< 3 days, Table 2). The DTR threshold above which RR risk increased varied between cities, but was roughly 20 °C in ORF, 22 °C in LYH, PHF, and RIC, and 25 °C in CHO. The lowest threshold was observed in ROA (16 °C), which is the coldest location in the study because of elevation. ROA and CHO also exhibited the longest lagged effects, extending to four days for very high DTRs. The location with the outlier pattern is IAD, where the very highest DTRs were associated with reduced mortality at all lags. However, IAD did exhibit elevated risk for short lags, albeit over a lower DTR range (17–23 °C). The DLNM results were used to identify high risk DTR days—days with elevated mortality and DTR that exceeds the threshold at lag day zero—and associated lags (Table 2 and outlined regions in Fig. 2). The lagged RR response at each DTR value at and above the various thresholds are presented for each city in Supplemental Fig. 1.

**Table 2:** Criteria used to identify high-DTR days for each location (see Fig. 2)

	CHO	IAD	LYH	ORF	PHF	RIC	ROA
Threshold DTR (°C)	≥ 25	17–23	≥ 22	≥ 20	≥ 22	≥ 22	≥ 16
Frequency (%)	3 (0.1)	857 (14.7)	55 (0.9)	30 (0.5)	18 (0.3)	26 (0.4)	930 (15.9)
Lag (days)	0–4	0–1	0–2	0–1	0–1	0–2	0–1 (16–18 °C)
							0–2 (18–20 °C)
							0–3 (20–21 °C)
							0–4 (21–22 °C)
							0–5 (≥ 22 °C)

The sensitivity of the DLNMs to variable selection was examined using a consensus model (Eq. 3). The lagged relative risk patterns (Supplemental Fig. 2) are broadly comparable to the results presented in Fig. 2. This strongly suggests that the model is not overly sensitive to variable selection, given that so many of the temperature and humidity variables are highly correlated (Supplemental Table 4), and that the specific splines and control variables exert only a minor influence on the overall shape of the relationships across cities.

**Fig. 3:**
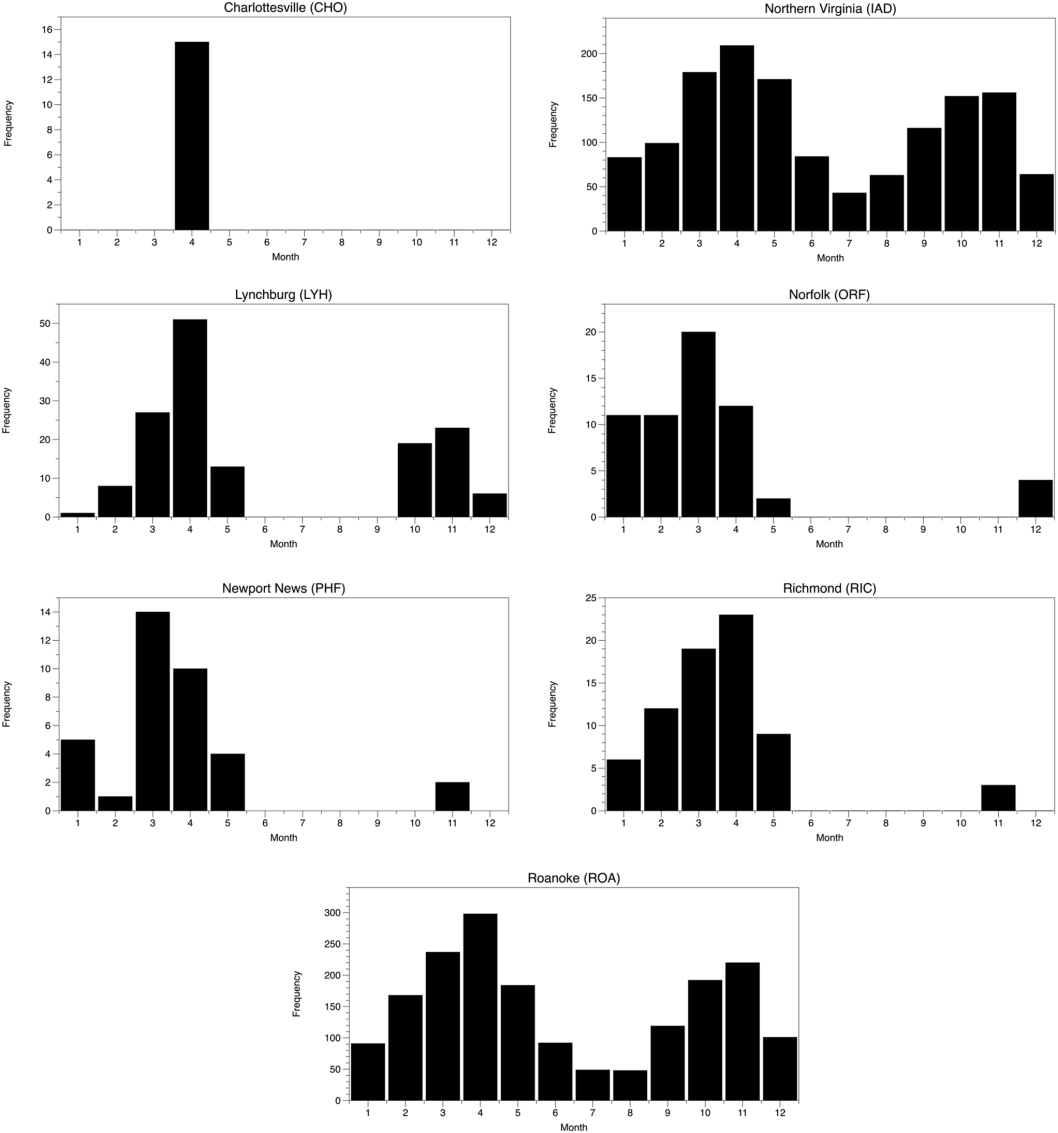
Monthly frequency of days with high mortality risk at each location

Days with high DTR primarily occur in the spring (Fig. 3). With the onset of spring, despite lengthening days and a declining solar noon zenith angle, cold Arctic air masses and the tropospheric polar vortex occur over the mid-Atlantic region, and strong frontal passages are common. Thus, days with substantial warm to cold transitions over 24 h do occur. As summer approaches, the cold pool of Arctic air is diminished and restricted by polar vortex contraction to higher latitudes. Furthermore, high summertime humidity raises the dew point temperature, which effectively limits the extent to which overnight temperatures can drop, even under strong anticyclonic conditions with light winds and extensive radiative cooling. Autumn (Sep–Nov) dew point temperatures are significantly higher than in spring (Mar–May), and the coldest air masses that reach Virginia are typically not below freezing until late October or November.

To explore further the climatology of high risk DTR days, we compared the frequency of SSC types on days that exceed the DTR threshold vs. all other days for each city using a chi-square analysis. High DTR days are characterized by an abundance of DM and DT synoptic types and an almost complete lack of moist (M) days (Table 3). In fact, for every location, there is a lower percentage of MP, MM, and MT weather types on high DTR days. Although the frequency of transition days, which includes frontal passages (Hondula and Davis 2011) is high, as would be expected on high DTR days, the primary difference is driven by DT air masses. Climatologically, although the advection of pure, desert air from the Desert Southwest into Virginia does occur, these events are rare. More commonly, DT conditions arise from strong subsidence under anticyclonic flow and an upper air ridge, often associated with westerly or northwesterly flow and subsidence along the eastern slopes of the Appalachians. The resulting clear skies allow for high afternoon temperatures, and the low dew points and lack of wind provide ideal conditions for overnight temperatures to fall dramatically.

**Table 3:** Spatial synoptic classification weather type frequencies (%) for days identified as high-DTR days (see Table 2; Fig. 2) vs. all other days, for each location. (no tests were completed for CHO because of the small sample of high-DTR days there.). A Pearson Chi-square test indicates that frequency distributions are significantly different for all locations (*p* < 0.001). DM = dry moderate; DP = dry polar; DT = dry tropical; MM = moist moderate; MP = moist polar; MT = moist tropical; TR = transition

	IAD	LYH	ORF	PHF	RIC	ROA
DM (DTR)	55	41	18	56	19	49
DM	30	35	30	33	32	30
DP (DTR)	8	8	7	0	0	8
DP	14	13	9	10	10	12
DT (DTR)	22	35	38	28	65	24
DT	3	5	6	6	8	5
MM (DTR)	0	3	2	0	0	1
MM	13	12	12	10	10	13
MP (DTR)	0	3	3	0	0	0
MP	7	6	4	4	5	7
MT (DTR)	5	7	10	0	0	8
MT	24	23	30	29	26	24
TR (DTR)	11	3	22	17	15	10
TR	10	7	9	9	9	9

To examine the weather associated with high DTR in additional detail, we compared weather variables for the DTR and non-DTR days by month using two-tailed, independent samples t-tests (Supplemental Table 5). Generally, high DTR days are characterized by cool, dry air (low humidity), and calm mornings with high pressure—all conditions associated with strong radiative cooling in which temperatures can drop markedly. Afternoons and evenings tend to be warm and dry. During the warm season in this humid subtropical climate, humidity is high, and the dew point temperature governs the extent to which overnight temperatures can decline (Davis et al. 2020). Thus, summer DTR is suppressed by the high humidity, so days with very large DTRs can only occur when humidity is low. In the cold season, however, cold and dry air masses are common, allowing for low overnight temperatures. This accounts for the lack of a significant DTR correlation with most thermal/moisture variables at 1 a.m. and 7 a.m. in winter.

We now examine the disease and demographic factors associated with high DTR days. In this analysis, we include all DTR days along with the associated lags, as indicated in Fig. 2; Table 2. We compare the mean mortality between high DTR days and all other days using one-tailed, independent samples t-tests based on 1000 bootstrapped samples. A one-tailed test was employed because we are not interested in unusually low mortality that might be linked to high DTR. High DTR is associated with at least one high mortality category at every location but CHO (which had a very small sample size) (Table 4). High circulatory or respiratory mortality was associated with high DTR in five of the remaining six locations. Six other disease categories had high DTR mortality at only one location. With respect to age, the most consistent responses were for age 50–64 and the elderly (age ≥ 75 years). There is no consistent mortality difference based on gender or race.

**Table 4:** Diseases and demographic groups with significantly higher mortality rates on high-DTR days for each location, based on one-tailed, independent sample t-tests (*p* < 0.05). Sample sizes of the “case” group are shown in the last row

	CHO	IAD	LYH	ORF	PHF	RIC	ROA
MDC 1			X				
MDC 4		X		X			X
MDC 5			X			X	
MDC 7					X		
MDC 9						X	
MDC 12				X			
MDC 19				X			
Cancer				X			
Age 12–17							X
Age 30–39					X		
Age 40–49			X				
Age 50–64						X	X
Age ≥ 75		X		X			X
Female		X					X
Male							X
Black				X		X	
White				X		X	X
Total				X			X
Case Group N		1419	148	60	36	72	205

## Discussion

The key findings are:

1. mortality risk increases on days with high DTR;
2. the lag effect is short (typically 0–2 days);
3. the models control for temperature, so the mortality increase is presumably associated with temperature *variability*;
4. dangerous DTR days are rare (occurring < 1% of the time in most cities) as the DTR thresholds required to trigger a mortality response are high;
5. very high DTR days primarily occur in spring on dry days dominated by high pressure; and,
6. high DTR impacts a wide range of disease types and demographic groups.

It is important to interpret the mortality linkage to DTR within a climatic context. On a large majority of days, the variation in temperature over the course of 24 h has no relation to mortality. A linkage is only evident on rare days with cold mornings and very warm afternoons that occur two or three times per year at most locations. Most of these days occur in the spring and autumn, which are typically times when mortality is transitioning between the winter peak and summer minimum. Nonetheless, there is evidence in Virginia of a positive mortality relationship that aligns with a number of other published studies at other locations (Kan et al. 2007; Lim et al. 2012, 2015; Cheng et al. 2014; Zhou et al. 2014; Ding et al. 2015, 2016; Kim et al. 2016; Lee et al. 2018a, b, 2019; Tang et al. 2018; Sharafkhani et al. 2019; Kai et al. 2023; Amoatey et al. 2024). However, most of these studies did not examine the climate associated with high DTR days.

The linkage of high DTR to circulatory and respiratory mortality is consistent with findings for other countries (Kan et al. 2007; Song et al. 2008; Cheng et al. 2014; Zhou et al. 2014; Ding et al. 2016; Kim et al. 2016; Lee et al. 2018b; Tang et al. 2018). As circulatory and respiratory disease are among the most common causes of death, these MDCs are frequently selected for analysis. Arguments have been posited that a lack of physiological acclimatization can impose a strain on the respiratory and circulatory systems (de Freitas and Grigorieva 2009; Davis and Enfield 2018). In climates with seasonal temperatures, the cold-to-hot and hot-to-cold transitions in spring and fall can impose thermal stresses on the body that manifest a physiological response. These responses can include, but are not limited to, variations in sweating rates and resting heart rates, blood pressure, resting metabolism, core temperature, etc. These changes typically transpire over the period of days to weeks (Williams et al. 1967; Hori 1978, 1995; Mathew et al. 1981; Armstrong and Maresh 1991; Saat et al. 2005; Kampmann et al. 2008; Estela 2018).

The general relationship between mortality and DTR, with high DTR thresholds and short lags, is very similar to the response to temperature and other thermal indices observed across the world (e.g., Kunst et al. 1993; Iñiguez et al. 2010; Guo et al. 2014; Ma et al. 2015). However, most DTR models (including those in this research) statistically control for temperature (and often humidity as well), thus indicating that the DTR response differs from the temperature response. One possible explanation is that the offending hot spring days identified in this study are imposing heat stress on unacclimatized individuals who have not yet adapted to warm conditions that have not been experienced for months. However, this would suggest that the DTR response is, in fact, a heat response, and that the observed low morning temperatures are unrelated to the mortality. If so, this raises the question of whether the modeling approach completely controls for temperature or whether it is possible that there remains some residual effect of temperature alone that is not accounted for in the models.

Of particular interest in this regard is the research by Lee et al. (2018b, 2019). In separate studies for locations in Japan, Korea, and Taiwan, they found that the DTR risk becomes stronger as temperatures increase. This result implies that the risk from DTR is related to heat stress, as cool evenings and mornings provide needed respite from the day’s hot conditions. Vicedo-Cabrera et al. (2016) examined mortality in six climatically different cities in the U.S. and Europe. Although they found positive associations between mortality and mean temperature, they found no consistent relationships to DTR and suggest that little is gained by adding this variable to the models.

A more detailed investigation of the influence of rapid weather changes on health, particularly in the spring and fall, is merited. The human respiratory system is uniquely impacted by these changes, as inhaled air is warmed and humidified before being exhaled, which imposes an energy cost to the body’s upper and lower respiratory tracts (Höppe 1981; de Freitas and Grigorieva 2009). Thus, stresses exerted by the external environment may result in respiratory strain, particularly among compromised individuals. For example, Vitkina et al. (2019) linked short-term weather changes to reduced pulmonary function and antioxidant defense in Russia. Certain respiratory diseases are associated with cardiac conditions (Rothnie et al. 2015), so there is a linkage between two of the most common causes of death. Davis and Enfield (2018) related lack of acclimatization to hospital admissions for respiratory disease in Charlottesville, Virginia. They found that transitions from warm and humid to cold and dry conditions, which typically occur in autumn, were linked to peak hospital admissions. However, that contrasts the results shown here for mortality and DTR, which has a pronounced peak in spring and only a weaker association in autumn for some locations.

It is also possible that some meteorological factor correlated with high DTR is responsible for high mortality events. A number of studies have identified linkages between short-term weather changes and mortality. Rapid pressure declines, associated with temperature increases, have been associated with elevated mortality in the Czech Republic (Plavcová and Kyselý 2010; Plavcová and Urban 2020). Similarly, in two U.S. studies, mortality spikes were associated with rapid pressure declines (Allen and Sheridan 2014), particularly when proceeded by a prolonged cold anomaly in winter (Allen and Lee 2014). In contrast, Törő et al. (2010) associated sudden cardiac death to rapid pressure increases in Budapest.

High DTR is also commonly associated with frontal passages, which have likewise been linked to increased mortality. Lee (2015) found that U.S. mortality tends to spike in conjunction with warm frontal passages, particularly when preceded by a string of anomalously cold and dry days. This is generally consistent with observations of day-to-day temperature changes and mortality in Virginia cities (Pane and Davis 2024). In Birmingham, England, ischemic heart disease mortality was most prominently associated with short term changes in dew point temperature (Mcgregor 2001). In Cape Town, South Africa, cardiovascular mortality tends to peak when the hot and dry Berg winds, a katabatic flow from the northern escarpment, are followed by a cold front passage (Heunis et al. 1995). In each of these cases, it is plausible that the human body is not responding to a single meteorological factor, but to the concurrent suite of weather conditions. Note, however, that frontal passages comprise a small percentage of high DTR days in Virginia (Table 3).

Some of the results should be considered with caution given the small sample size of DTR cases that exceed the threshold in most cities. The DTR effect is most evident at the margin of the distribution, where model fitting can be imprecise. A bootstrapping procedure was employed to help mitigate the sample size issue. Likewise, there is always a concern for false positives, and this likely is the case for some of the MDC results that were only observed for a single location. We do not know the exposure of each decedent, and local microclimatic variability can have some influence. The use of a single weather station to represent a multi-county region has inherent error, and we made every effort to choose locations that are climatologically representative. Employing more stations to improve spatial coverage would result in smaller daily sample sizes and reduced statistical robustness, which motivated us to emphasize the more populated metropolitan areas in this research. The extent to which the results for this population in Virginia might be generalizable to other populations and locations is uncertain. More research is needed to examine DTR and mortality for other midlatitude climates to determine if springtime DTR extremes are associated with high mortality at short lags.

## Conclusions

There is a direct relationship between days with large temperature change and human mortality in Virginia. Mortality relative risk varies from 1.02 to 1.20 and typically is highest 1–2 days after the diurnal temperature range exceeds 20 °C, with the critical threshold varying by location. High DTR days predominantly occur in the spring season and are associated with dry air, high pressure, and light winds—conditions that support radiative cooling with low overnight temperatures and warm and sunny afternoons. DTR-related deaths tend to arise from circulatory or respiratory conditions and are most common among decedents in the 50–64 and ≥ 75 year age ranges.

It is important to remember that, at least based on this population, DTR-related deaths are rare events, typically occurring less than 1% of the time. The climate conditions required to produce 24-hour temperature changes that exceed the threshold do not occur routinely. Furthermore, they almost always occur in the spring and autumn, at times when the weather is most changeable. Nevertheless, when these rare events do occur, the related mortality can increase by as much as 20% above the baseline rate.

The lack of a consistent physiological response (in terms of an MDC that is systematically higher in association with high DTRs) leaves the physiological explanation of the relationship unsettled. A case must be made that certain individuals are physically unable to cope with short-term temperature *changes*—changes that are of themselves unrelated to hot or cold conditions—and that this inability to adapt compromises one or more critical bodily functions. At this time, these relationships are correlative, and more work on the physiological responses is required. It is likewise important to understand the climatic context in which high DTR events occur, and more work is needed to characterize the weather conditions on the days linked to these high mortality events. Without additional, convincing evidence that human health is impacted by 24-hour weather variability, the DTR-human health enigma remains unresolved.

## Electronic supplementary material

Below is the link to the electronic supplementary material.

Supplementary Material 1

## Data Availability

Access to the mortality data used in this study is governed by the Virginia Department of Health (VDH)’s and the University of Virginia (UVA)’s Institutional Review Board (IRB) Data Management and Security Plan, which is available from the authors upon request. After the study has ended, anonymized datasets can be made available to other researchers, with the agreement of the authors and both IRBs, upon request. The research team will decide upon the types of data that may be made available to others, in compliance with the HIPAA laws of the United States of America and the VDH and UVA IRB policies on data security.

## Code availability

Analysis was completed using customized R code and SPSS packaged routines and can be made available upon request.

## References

[CR1] Allen MJ, Lee CC (2014) Investigating high mortality during the cold season: mapping mean weather patterns of temperature and pressure. Theoret Appl Climatol 118:419–428

[CR2] Allen MJ, Sheridan SC (2014) High-mortality days during the winter season: comparing meteorological conditions across 5 US cities. Int J Biometeorol 58:217–225 23417344 10.1007/s00484-013-0640-4

[CR3] Amoatey P, Osborne NJ, Darssan D, Xu Z, Doan QV, Phung D (2024) The effects of diurnal temperature range on mortality and emergency department presentations in Victoria state of Australia: a time-series analysis. Environ Res 240:117397 37879389 10.1016/j.envres.2023.117397

[CR4] Anderson GB, Bell ML, Peng RD (2013) Methods to calculate the heat index as an exposure metric in environmental health research. Environ Health Perspect 121(10):1111–1119 23934704 10.1289/ehp.1206273 PMC3801457

[CR5] Armstrong LE, Maresh CM (1991) The induction and decay of heat acclimatisation in trained athletes. Sports Med 12(5):302–312 1763248 10.2165/00007256-199112050-00003

[CR6] Bonan GB (2001) Observational evidence for reduction of daily maximum temperature by croplands in the Midwest United States. J Clim 14:2430–2442

[CR7] Braganza K, Karoly DJ, Arblaster JM (2004) Diurnal temperature range as an index of global climate change during the twentieth century. Geophys Res Lett 31:L13217

[CR8] Brook RD, Weder AB, Rajagopalan S (2011) Environmental hypertensionology the effects of environmental factors on blood pressure in clinical practice and research. J Clin Hypertens 13(11):836–842 10.1111/j.1751-7176.2011.00543.x PMC8108751 22051429

[CR9] Buguet A (2007) Sleep under extreme environments: effects of heat and cold exposure, altitude, hyperbaric pressure and microgravity in space. J Neurol Sci 262(1–2):145–152 17706676 10.1016/j.jns.2007.06.040

[CR10] Cheng J, Xu Z, Zhu R, Wang X, Jin L, Song J, Su H (2014) Impact of diurnal temperature range on human health: a systematic review. InternationalJjournal Biometeorol 58:2011–2024 10.1007/s00484-014-0797-5 24535132

[CR12] Davis RE, Enfield KB (2018) Respiratory hospital admissions and weather changes: a retrospective study in Charlottesville, Virginia, USA. Int J Biometeorol 62:1015–1025 29417216 10.1007/s00484-018-1503-9

[CR11] Davis RE, McGregor GR, Enfield KB (2016) Humidity: a review and primer on atmospheric moisture and human health. Environ Res 144:106–116 26599589 10.1016/j.envres.2015.10.014

[CR13] Davis RE, Hondula DM, Sharif H (2020) Examining the diurnal temperature range enigma: why is human health related to the daily change in temperature? Int J Biometeorol 64(3):397–407 31720855 10.1007/s00484-019-01825-8

[CR14] Davis RE, Roney PC, Pane MM, Johnson MC, Leigh HV, Basener W, Curran AL, DeMarcy B, Jang J, Schroeder C, DeGuzman PB, Novicoff WM (2023) Climate and human mortality in Virginia, 2005–2020. Science of The Total Environment, 894, 164825 10.1016/j.scitotenv.2023.164825 37343846

[CR15] De Freitas CR, Grigorieva EA (2009) The acclimatization thermal strain index (ATSI): a preliminary study of the methodology applied to climatic conditions of the Russian Far East. Int J Biometeorol 53(4):307–315. 10.1007/s00484-009-0215-6 19238456 10.1007/s00484-009-0215-6

[CR16] Ding Z, Guo P, Xie F, Chu H, Li K, Pu J et al (2015) Impact of diurnal temperature range on mortality in a high plateau area in southwest China: a time series analysis. Sci Total Environ 526:358–365 25962628 10.1016/j.scitotenv.2015.05.012

[CR17] Ding Z, Li L, Xin L, Pi F, Dong W, Wen Y et al (2016) High diurnal temperature range and mortality: effect modification by individual characteristics and mortality causes in a case-only analysis. Sci Total Environ 544:627–634 26674692 10.1016/j.scitotenv.2015.12.016

[CR18] Durre I, Wallace JM The warm season dip in the diurnal temperature range over the eastern United States. J. Climate 14:354–360.

[CR72] Easterling DR, Horton B, Jones PD, Peterson TC, Karl TR, Parker DE, Salinger MJ, Razuvayev V, Plummer N, Jamason P, Folland CK (1997) Maximum and minimum temperature trends for the globe. Science 277(5324):364–367.

[CR19] Estela LBL (2018) Biometeorological forecasts for health surveillance and prevention of meteor-tropic effects. Int J Biometeorol 62(5):741–771 28905125 10.1007/s00484-017-1405-2

[CR20] Gallo KP, Easterling DR, Peterson TC The influence of land use/land cover on climatological values of the diurnal temperature range. J Climate 9:2941–2944.

[CR73] Gasparrini A (2011) Distributed lag linear and non-linear models in R: the package dlnm. J Statist Softw 43(8):1. PMC3191524 22003319

[CR21] Geerts B (2003) Empirical estimation of the monthly-mean daily temperature range. Theoret Appl Climatol 73:101–132

[CR22] Graudenz GS, Landgraf RG, Jancar S, Tribess A, Fonseca SG, Faé KC, Kalil J (2006) The role of allergic rhinitis in nasal responses to sudden temperature changes. J Allergy Clin Immunol 118(5):1126–1132 17088139 10.1016/j.jaci.2006.07.005

[CR23] Guo Y, Gasparrini A, Armstrong B, Li S, Tawatsupa B, Tobias A et al (2014) Global variation in the effects of ambient temperature on mortality: a systematic evaluation. Epidemiology 25(6):781–789 25166878 10.1097/EDE.0000000000000165 PMC4180721

[CR24] Heunis JC, Olivier J, Bourne DE (1995) Short-term relationships between winter temperatures and cardiac disease mortality in Cape Town. South Afr Med J, 85(10) 8596965

[CR26] Hondula DM, Davis RE (2011) Climatology of winter transition days for the contiguous USA, 1951–2007. Theoret Appl Climatol 103:27–37

[CR25] Höppe P (1981) Temperatures of expired air under varying climatic conditions. Int J Biometeorol 25(2):127–132. 10.1007/BF02184460 7251219 10.1007/BF02184460

[CR27] Hori S (1978) Index for the assessment of heat tolerance. J Hum Ergol 7(2):135–144 756447

[CR28] Hori S (1995) Adaptation to heat. Jpn J Physiol 45(6):921–946. 10.2170/jjphysiol.45.921 8676578 10.2170/jjphysiol.45.921

[CR29] Iñiguez C, Ballester F, Ferrandiz J, Pérez-Hoyos S, Sáez M, López A (2010) Relation between temperature and mortality in thirteen Spanish cities. Int J Environ Res Public Health 7(8):3196–3210 20948955 10.3390/ijerph7083196 PMC2954576

[CR30] Jackson LS, Forster PM (2010) An empirical study of geographic and seasonal variations in diurnal temperature range. J Clim 23:3205–3221

[CR31] Kai X, Hong Z, Hong Y, Wang X, Li C (2023) Short-term impact of diurnal temperature range on cardiovascular diseases mortality in residents in northeast China. Sci Rep 13(1):11037 37419976 10.1038/s41598-023-38129-2 PMC10328923

[CR33] Kampmann B, Brode P, Schutte M et al (2008) Lowering of resting core temperature during acclimation is influenced by exercise stimulus. Eur J Appl Physiol 104:321–327 18193268 10.1007/s00421-007-0658-6

[CR32] Kan H, London SJ, Chen H, Song G, Chen G, Jiang L et al (2007) Diurnal temperature range and daily mortality in Shanghai, China. Environ Res 103(3):424–431 17234178 10.1016/j.envres.2006.11.009

[CR34] Karl TR, Jones PD, Knight RW, Kukla G, Plummer N, Razuvaye V et al (1993) A new perspective on recent global warming: asymmetric trends of daily maximum and minimum temperature. Bull Am Meteorol Soc 74(6):1007–1024

[CR35] Keatinge WR, Coleshaw SR, Easton JC, Cotter F, Mattock MB, Chelliah R (1986) Increased platelet and red cell counts, blood viscosity, and plasma cholesterol levels during heat stress, and mortality from coronary and cerebral thrombosis. Am J Med 81(5):795–800 3776986 10.1016/0002-9343(86)90348-7

[CR36] Kim J, Shin J, Lim YH, Honda Y, Hashizume M, Guo YL et al (2016) Comprehensive approach to understand the association between diurnal temperature range and mortality in East Asia. Sci Total Environ 539:313–321 26363726 10.1016/j.scitotenv.2015.08.134

[CR37] Kunst AE, Looman CW, Mackenbach JP (1993) Outdoor air temperature and mortality in the Netherlands: a time-series analysis. Am J Epidemiol 137(3):331–341 8452141 10.1093/oxfordjournals.aje.a116680

[CR38] Leathers DJ, Palecki MA, Robinson RA, Dewey KF (1998) Climatology of the daily temperature range annual cycle in the United States. Clim Res 9:197–211

[CR39] Lee CC (2015) A systematic evaluation of the lagged effects of spatiotemporally relative surface weather types on wintertime cardiovascular-related mortality across 19 US cities. Int J Biometeorol 59:1633–1645 25711484 10.1007/s00484-015-0970-5

[CR40] Lee W, Bell ML, Gasparrini A, Armstrong BG, Sera F, Hwang S et al (2018a) Mortality burden of diurnal temperature range and its temporal changes: a multi-country study. Environ Int 110:123–130 29089167 10.1016/j.envint.2017.10.018

[CR41] Lee W, Kim Y, Honda Y, Kim H (2018b) Association between diurnal temperature range and mortality modified by temperature in Japan, 1972–2015: investigation of spatial and temporal patterns for 12 cause-specific deaths. Environ Int 119:379–387 30005186 10.1016/j.envint.2018.06.020

[CR42] Lee W, Chung Y, Choi HM, Kim D, Honda Y, Guo YLL, Kim H (2019) Interactive effect of diurnal temperature range and temperature on mortality, northeast Asia. Epidemiology 30:S99–S106 31181012 10.1097/EDE.0000000000000997

[CR43] Lim YH, Park AK, Kim H (2012) Modifiers of diurnal temperature range and mortality association in six Korean cities. Int J Biometeorol 56:33–42 21207069 10.1007/s00484-010-0395-0

[CR44] Lim YH, Reid CE, Mann JK, Jerrett M, Kim H (2015) Diurnal temperature range and short-term mortality in large US communities. Int J Biometeorol 59:1311–1319 25465402 10.1007/s00484-014-0941-2

[CR45] Ma W, Wang L, Lin H, Liu T, Zhang Y, Rutherford S, Zhou M (2015) The temperature–mortality relationship in China: an analysis from 66 Chinese communities. Environ Res 137:72–77 25490245 10.1016/j.envres.2014.11.016

[CR46] Masterton JM, Richardson FA (1981) Humidex: a method of quantifying human discomfort due to excessive heat and humidity. CLI 1–79. Environment Canada, Atmospheric Environment Service Downsview, Ontario

[CR47] Mathew L, Purkayastha SS, Jayashankar A, Nayar HS (1981) Physiological characteristics of cold acclimatization in man. Int J Biometeorol 25(3):191–198. 10.1007/BF02184518 7275347 10.1007/BF02184518

[CR48] McGregor GR (2001) The meteorological sensitivity of ischaemic heart disease mortality events in Birmingham, UK. Int J Biometeorol 45:133–142 11594633 10.1007/s004840100094

[CR49] Mercer JB, Østerud B, Tveita T (1999) The effect of short-term cold exposure on risk factors for cardiovascular disease. Thromb Res 95(2):93–104 10418798 10.1016/s0049-3848(99)00028-6

[CR50] Normand C (1946) Energy in the atmosphere. Q J Roy Met Soc 72(312–313):145–167

[CR51] Osczevski R, Bluestein M (2005) The new wind chill equivalent temperature chart. Bull Am Meteorol Soc 86(10):1453–1458

[CR52] Pane MM, Davis RE (2024) The association between short-term temperature variability and mortality in Virginia. PLoS ONE, 19(9), e0310545 10.1371/journal.pone.0310545 PMC11414919 39302917

[CR53] Plavcová E, Kyselý J (2010) Relationships between sudden weather changes in summer and mortality in the Czech Republic, 1986–2005. Int J Biometeorol 54:539–551 20169367 10.1007/s00484-010-0303-7

[CR54] Plavcová E, Urban A (2020) Intensified impacts on mortality due to compound winter extremes in the Czech Republic. Sci Total Environ 746:141033 32750577 10.1016/j.scitotenv.2020.141033

[CR55] Powell EJ, Keim BD (2015) Trends in daily temperature and precipitation extremes for the southeastern United States: 1948–2012. J Clim 28:1592–1612

[CR56] Qu M, Wan J, Hao X (2014) Analysis of diurnal air temperature range change in the continental United States. Weather Clim Extrem 4:86–95

[CR57] Rothnie KJ, Yan R, Smeeth L, Quint JK (2015) Risk of myocardial infarction (MI) and death following MI in people with chronic obstructive pulmonary disease (COPD): a systematic review and meta-analysis. BMJ Open 5(9):e007824. 10.1136/bmjopen-2015-007824 10.1136/bmjopen-2015-007824 PMC4567661 26362660

[CR58] Saat M, Sirisinghe RG, Singh R, Tochihara Y (2005) Effects of short- term exercise in the heat on thermoregulation, blood parameters, sweat secretion and sweat composition of tropic-dwelling subjects. J Physiol Anthropol Appl Hum Sci 24(5):541–549. 10.2114/jpa.24.541 10.2114/jpa.24.541 16237263

[CR59] Scheitlin KN, Dixon PG Diurnal temperature range variability due to land cover and airmass types in the southeast. J. Appl. Meteor. Climatol., 49, 879–888.

[CR74] Sharafkhani R, Khanjani N, Bakhtiari B, Jahani Y, Tabrizi JS, Tabrizi FM (2019) Diurnal temperature range and mortality in Tabriz (the northwest of Iran). Urban Clim 27:204–211.

[CR60] Sheridan SC (2002) The redevelopment of a weather-type classification scheme for North America. Int J Climatology: J Royal Meteorological Soc 22(1):51–68

[CR61] Song G, Chen G, Jiang L, Zhang Y, Zhao N, Chen B, Kan H (2008) Diurnal temperature range as a novel risk factor for COPD death. Respirology 13(7):1066–1069 18922144 10.1111/j.1440-1843.2008.01401.x

[CR62] Sun D, Pinker R, Kafatos M (2006) Diurnal temperature range over the United States: a satellite view. Geophys Res Lett, 33; L05705.

[CR71] Sun X, Ren G, You Q, Ren Y, Xu W, Xue X, Zhan Y, Zhang S, Zhang P (2019) Global diurnal temperature range (DTR) changes since 1901. Climate Dynamics 52; 3343–3356

[CR63] Tang J, Xiao CC, Li YR, Zhang JQ, Zhai HY, Geng XY et al (2018) Effects of diurnal temperature range on mortality in Hefei city, China. Int J Biometeorol 62:851–860 29224119 10.1007/s00484-017-1486-y

[CR64] Thom EC (1959) The discomfort index. Weatherwise 12(2):57–60

[CR65] Törő K, Bartholy J, Pongrácz R, Kis Z, Keller É, Dunay G (2010) Evaluation of meteorological factors on sudden cardiovascular death. J Forensic Leg Med 17(5):236–242 20569948 10.1016/j.jflm.2010.02.008

[CR66] Vicedo-Cabrera AM, Forsberg B, Tobias A, Zanobetti A, Schwartz J, Armstrong B, Gasparrini A (2016) Associations of inter-and intraday temperature change with mortality. Am J Epidemiol 183(4):286–293 26811244 10.1093/aje/kwv205 PMC4753281

[CR67] Vitkina TYI, Veremchuk LV, Mineeva EE, Gvozdenko TYA, Antonyuk MV, Novgorodtseva TYP, Grigorieva EA (2019) The influence of weather and climate on patients with respiratory diseases in Vladivostok as a global health implication. J Environ Health Sci Eng 17(2):907–916 32030162 10.1007/s40201-019-00407-5 PMC6985342

[CR68] Vose RS, Easterling DR, Gleason B (2005) Maximum and minimum temperature trends for the globe: an update through 2004. Geophys Res Lett 32:L23822

[CR69] Williams CG, Wyndham CH, Morrison JF (1967) Rate of loss of acclimatization in summer and winter. J Appl Physiol 22(1):21–26. 10.1152/jappl.1967.22.1.21 6017648 10.1152/jappl.1967.22.1.21

[CR70] Zhou X, Zhao A, Meng X, Chen R, Kuang X, Duan X, Kan H (2014) Acute effects of diurnal temperature range on mortality in 8 Chinese cities. Sci Total Environ 493:92–97 24937494 10.1016/j.scitotenv.2014.05.116

