# Supplementary material for: Mortality and diurnal temperature range in Virginia
Source: Int J Biometeorol. 2025 Jan 30;69(4):725–38. doi: 10.1007/s00484-025-02850-6 (PMC11946996; doi:10.1007/s00484-025-02850-6)
Supplement: Supplementary file 1 — Supplementary Material 1 [file 484_2025_2850_MOESM1_ESM.docx]

**Supplemental Tables**

Supplemental Table 1. Diseases associated with each Major Diagnostic Category (MDC).

| **MDC** | **Description** |
| --- | --- |
| 1 | Diseases and Disorders of the Nervous System |
| 2 | Diseases and Disorders of the Eye |
| 3 | Diseases and Disorders of the Ear, Nose, and Throat |
| 4 | Diseases and Disorders of the Respiratory System |
| 5 | Diseases and Disorders of the Circulatory System |
| 6 | Diseases and Disorders of the Digestive System |
| 7 | Diseases and Disorders of the Hepatobillary System and Pancreas |
| 8 | Diseases and Disorders of the Musculoskeletal System and Connective Tissue |
| 9 | Diseases and Disorders of the Skin, Subcutaneous Tissue, and Breast |
| 10 | Diseases and Disorders of the Endocrine, Nutritional, and Metabolic System |
| 11 | Diseases and Disorders of the Kidney and Urinary Tract |
| 12 | Diseases and Disorders of the Male Reproductive System |
| 13 | Diseases and Disorders of the Female Reproductive System |
| 14 | Pregnancy, Childbirth, and Puerperium |
| 15 | Newborn and Other Neonates (Perinatal Period) |
| 16 | Diseases and Disorders of the Blood and Blood-forming Organs and Immunological Disorders |
| 17 | Myeloproliferative Diseases and Disorders (Poorly Differentiated Neoplasms) |
| 18 | Infectious and Parasitic Diseases and Disorders (Systemic or Unspecified Sites) |
| 19 | Mental Diseases and Disorders |
| 20 | Alcohol/Drug Use or Induced Mental Disorders |
| 21 | Injuries, Poison, and Toxic Effect of Drugs |
| 22 | Burns |
| 23 | Factors Influencing Health Status and Other Contacts with Health Services |
| 24 | Multiple Significant Trauma |
| 25 | Human Immunodeficiency Virus Infection |

Supplemental Table 2. Virginia counties and independent cities associated with each of the seven study regions and associated mortality.

| **CODE** | **LOCATION** | **COUNTIES** | **INDEPENDENT CITIES** | **TOTAL**  **DEATHS** | **MEAN DAILY DEATHS** |
| --- | --- | --- | --- | --- | --- |
| IAD | Northern Virginia | Arlington, Fairfax, Fauquier, Loudon, Prince William | Alexandria, Fairfax, Falls Church | 162,390 | 27.8 |
| CHO | Charlottesville | Albemarle, Culpeper, Fluvanna, Greene, Louisa, Madison, Nelson, Orange | Charlottesville | 32,073 | 5.5 |
| LYH | Lynchburg | Amherst, Appomattox, Bedford, Buckingham, Campbell, Charlotte, Prince Edward | Bedford, Lynchburg | 49,220 | 8.4 |
| PHF | Newport News | Accomack, Gloucester, James City, Northhampton, Northumberland, Lancaster, Mathews, Middlesex, York | Hampton, Newport News, Poquoson, Williamsburg | 88,691 | 15.2 |
| ORF | Norfolk | Isle of Wight, Surry | Chesapeake, Norfolk, Portsmouth, Suffolk, Virginia Beach | 137,238 | 23.5 |
| RIC | Richmond | Amelia, Charles City, Chesterfield, Cumberland, Goochland, Hanover, Henrico, King William, New Kent, Powhatan, Prince George | Colonial Heights, Hopewell, Petersburg, Richmond | 155,466 | 26.6 |
| ROA | Roanoke | Alleghany, Botetourt, Craig, Floyd, Giles, Montgomery, Pulaski | Roanoke, Salem | 74,501 | 12.7 |

Supplemental Table 3. Comprehensive list of variables used in the study.

Maximum temperature

Minimum temperature

Diurnal temperature range

(The following variables were extracted at 1 a.m., 7 a.m., 1 p.m., and 7 p.m. Eastern Standard Time)

Temperature

Wet bulb temperature

Dew point temperature

Relative humidity

Wind speed

Sea-level pressure

(These variables were computed at 1 a.m., 7 a.m., 1 p.m., and 7 p.m. Eastern Standard Time)

Apparent temperature

Temperature-humidity index

Humidex

Wind chill index

(Nominal variables that served as possible predictors)

Day of week

Holidays (Thanksgiving and Christmas)

Winter weather (at least 4 inches of snow accumulated on one or two consecutive days or observations of freezing rain or freezing drizzle)

(Air quality varibles)

Ozone

PM_2.5_

Supplemental Table 4. Pearson product correlation of the thermal/moisture variables at 1 p.m. local time for each city. T=temperature, T_w_=wet bulb temperature, T_d_=dew point temperature, AT=apparent temperature, THI=temperature-humidity index, Hx=humidex.

| **CHO** | *T* | *T_w_* | *T_d_* | *RH* | *AT* | *THI* | *Hx* |
| --- | --- | --- | --- | --- | --- | --- | --- |
| *T* | 1 |  |  |  |  |  |  |
| *T_w_* | .978 | 1 |  |  |  |  |  |
| *T_d_* | .830 | .928 | 1 |  |  |  |  |
| *RH* | –.047 | .158 | .507 | 1 |  |  |  |
| AT | .994 | .983 | .853 | –.002 | 1 |  |  |
| *THI* | .995 | .995 | .884 | .055 | .994 | 1 |  |
| *Hx* | .983 | .995 | .905 | .109 | .991 | .994 | 1 |

| **IAD** | *T* | *T_w_* | *T_d_* | *RH* | *AT* | *THI* | *Hx* |
| --- | --- | --- | --- | --- | --- | --- | --- |
| *T* | 1 |  |  |  |  |  |  |
| *T_w_* | .984 | 1 |  |  |  |  |  |
| *T_d_* | .867 | .943 | 1 |  |  |  |  |
| *RH* | –.021 | .156 | .468 | 1 |  |  |  |
| AT | .994 | .987 | .887 | .027 | 1 |  |  |
| *THI* | .996 | .996 | .996 | .068 | .995 | 1 |  |
| *Hx* | .986 | .995 | .995 | .114 | .994 | .995 | 1 |

| **LYH** | *T* | *T_w_* | *T_d_* | *RH* | *AT* | *THI* | *Hx* |
| --- | --- | --- | --- | --- | --- | --- | --- |
| *T* | 1 |  |  |  |  |  |  |
| *T_w_* | .963 | 1 |  |  |  |  |  |
| *T_d_* | .856 | .944 | 1 |  |  |  |  |
| *RH* | –.027 | .199 | .481 | 1 |  |  |  |
| AT | .996 | .972 | .877 | .018 | 1 |  |  |
| *THI* | .991 | .990 | .907 | .084 | .993 | 1 |  |
| *Hx* | .985 | .984 | .921 | .117 | .992 | .994 | 1 |

| **ORF** | *T* | *T_w_* | *T_d_* | *RH* | *AT* | *THI* | *Hx* |
| --- | --- | --- | --- | --- | --- | --- | --- |
| *T* | 1 |  |  |  |  |  |  |
| *T_w_* | .986 | 1 |  |  |  |  |  |
| *T_d_* | .885 | .951 | 1 |  |  |  |  |
| *RH* | .014 | .179 | .470 | 1 |  |  |  |
| AT | .992 | .986 | .901 | .060 | 1 |  |  |
| *THI* | .996 | .996 | .921 | .097 | .993 | 1 |  |
| *Hx* | .985 | .996 | .939 | .154 | .993 | .994 | 1 |

| **PHF** | *T* | *T_w_* | *T_d_* | *RH* | *AT* | *THI* | *Hx* |
| --- | --- | --- | --- | --- | --- | --- | --- |
| *T* | 1 |  |  |  |  |  |  |
| *T_w_* | .986 | 1 |  |  |  |  |  |
| *T_d_* | .887 | .952 | 1 |  |  |  |  |
| *RH* | .066 | .231 | .512 | 1 |  |  |  |
| AT | .992 | .987 | .905 | .116 | 1 |  |  |
| *THI* | .996 | .996 | .923 | .150 | .993 | 1 |  |
| *Hx* | .985 | .995 | .941 | .206 | .993 | .994 | 1 |

| **RIC** | *T* | *T_w_* | *T_d_* | *RH* | *AT* | *THI* | *Hx* |
| --- | --- | --- | --- | --- | --- | --- | --- |
| *T* | 1 |  |  |  |  |  |  |
| *T_w_* | .983 | 1 |  |  |  |  |  |
| *T_d_* | .862 | .941 | 1 |  |  |  |  |
| *RH* | –.016 | .166 | .484 | 1 |  |  |  |
| AT | .993 | .986 | .885 | .038 | 1 |  |  |
| *THI* | .996 | .996 | .905 | .076 | .994 | 1 |  |
| *Hx* | .984 | .995 | .926 | .133 | .993 | .994 | 1 |

| **ROA** | *T* | *T_w_* | *T_d_* | *RH* | *AT* | *THI* | *Hx* |
| --- | --- | --- | --- | --- | --- | --- | --- |
| *T* | 1 |  |  |  |  |  |  |
| *T_w_* | .962 | 1 |  |  |  |  |  |
| *T_d_* | .856 | .942 | 1 |  |  |  |  |
| *RH* | –.054 | .172 | .458 | 1 |  |  |  |
| AT | .995 | .969 | .878 | –.007 | 1 |  |  |
| *THI* | .991 | .990 | .906 | .055 | .992 | 1 |  |
| *Hx* | .986 | .983 | .919 | .087 | .993 | .994 | 1 |

Supplemental Table 5. Summary of t-test results comparing weather variables on high DTR days (as identified in Figure 2) vs. all other days, by month, at each location, as noted in the top left cell. (CHO is not included given the lack of cases.) An upward-pointing arrow indicates that variable is statistically significantly higher on days with high DTR (p ≤ 0.05, two-tailed test); a downward arrow indicates a lower value on high DTR days. T = air temperature, T_w_ = wet bulb temperature, T_d_ = dew point temperature, RH = relative humidity, V = wind speed, SLP = sea-level pressure.

| **IAD** |  | **Jan** | **Feb** | **Mar** | **Apr** | **May** | **Jun** | **Jul** | **Aug** | **Sep** | **Oct** | **Nov** | **Dec** |
| --- | --- | --- | --- | --- | --- | --- | --- | --- | --- | --- | --- | --- | --- |
| **1 am** | *T* |  |  |  | ⇓ | ⇓ | ⇓ | ⇓ | ⇓ | ⇓ | ⇓ | ⇓ |  |
|  | *T_w_* |  |  |  | ⇓ | ⇓ | ⇓ | ⇓ | ⇓ | ⇓ | ⇓ | ⇓ |  |
|  | *T_d_* |  |  |  | ⇓ | ⇓ | ⇓ | ⇓ | ⇓ | ⇓ | ⇓ | ⇓ |  |
|  | *RH* |  |  |  |  |  |  |  |  |  | ⇑ | ⇑ |  |
|  | *V* |  | ⇓ | ⇓ | ⇓ | ⇓ | ⇓ | ⇓ | ⇓ | ⇓ | ⇓ | ⇓ |  |
|  | *SLP* |  | ⇑ | ⇑ | ⇑ | ⇑ | ⇑ | ⇑ | ⇑ | ⇑ | ⇑ | ⇑ |  |
|  |  |  |  |  |  |  |  |  |  |  |  |  |  |
| **7 am** | *T* |  |  | ⇓ | ⇓ | ⇓ | ⇓ | ⇓ | ⇓ | ⇓ | ⇓ | ⇓ |  |
|  | *T_w_* |  |  |  | ⇓ | ⇓ | ⇓ | ⇓ | ⇓ | ⇓ | ⇓ | ⇓ |  |
|  | *T_d_* |  |  |  | ⇓ | ⇓ | ⇓ | ⇓ | ⇓ | ⇓ | ⇓ | ⇓ |  |
|  | *RH* |  |  | ⇑ | ⇑ | ⇑ |  |  |  | ⇑ | ⇑ | ⇑ |  |
|  | *V* |  |  | ⇓ | ⇓ | ⇓ | ⇓ | ⇓ | ⇓ | ⇓ | ⇓ | ⇓ |  |
|  | *SLP* |  |  |  | ⇑ | ⇑ | ⇑ | ⇑ | ⇑ | ⇑ | ⇑ | ⇑ |  |
|  |  |  |  |  |  |  |  |  |  |  |  |  |  |
| **1 pm** | *T* | ⇑ | ⇑ | ⇑ | ⇑ | ⇑ |  | ⇑ | ⇑ | ⇑ | ⇑ | ⇑ | ⇑ |
|  | *T_w_* | ⇑ | ⇑ | ⇑ | ⇑ |  |  |  |  | ⇑ | ⇑ | ⇑ | ⇑ |
|  | *T_d_* |  |  |  | ⇓ | ⇓ | ⇓ | ⇓ | ⇓ | ⇓ |  |  |  |
|  | *RH* | ⇓ | ⇓ | ⇓ | ⇓ | ⇓ | ⇓ | ⇓ | ⇓ | ⇓ | ⇓ | ⇓ | ⇓ |
|  | *V* | ⇑ | ⇑ | ⇑ |  |  |  |  |  | ⇓ |  |  |  |
|  | *SLP* |  |  |  | ⇑ | ⇑ | ⇑ |  | ⇑ | ⇑ | ⇑ | ⇑ |  |
|  |  |  |  |  |  |  |  |  |  |  |  |  |  |
| **7 pm** | *T* | ⇑ | ⇑ | ⇑ | ⇑ | ⇑ |  | ⇑ | ⇑ | ⇑ | ⇑ | ⇑ | ⇑ |
|  | *T_w_* | ⇑ | ⇑ | ⇑ | ⇑ | ⇑ |  | ⇑ |  | ⇑ | ⇑ | ⇑ | ⇑ |
|  | *T_d_* |  |  |  |  | ⇓ |  | ⇓ | ⇓ | ⇓ |  |  |  |
|  | *RH* | ⇓ | ⇓ | ⇓ | ⇓ | ⇓ | ⇓ | ⇓ | ⇓ | ⇓ | ⇓ | ⇓ | ⇓ |
|  | *V* |  |  |  |  |  |  |  |  |  | ⇓ |  |  |
|  | *SLP* |  |  | ⇑ | ⇑ | ⇑ | ⇑ |  |  |  |  | ⇑ |  |
|  |  |  |  |  |  |  |  |  |  |  |  |  |  |
|  |  |  |  |  |  |  |  |  |  |  |  |  |  |

| **LYH** |  | **Jan** | **Feb** | **Mar** | **Apr** | **May** | **Jun** | **Jul** | **Aug** | **Sep** | **Oct** | **Nov** | **Dec** |
| --- | --- | --- | --- | --- | --- | --- | --- | --- | --- | --- | --- | --- | --- |
| **1 am** | *T* |  | ⇑ |  |  |  |  |  |  |  | ⇓ |  |  |
|  | *T_w_* |  |  |  |  | ⇓ |  |  |  |  | ⇓ |  |  |
|  | *T_d_* |  | ⇑ |  |  | ⇓ |  |  |  |  | ⇓ |  |  |
|  | *RH* |  |  | ⇓ | ⇓ | ⇓ |  |  |  |  |  |  |  |
|  | *V* |  |  |  | ⇓ | ⇓ |  |  |  |  | ⇓ | ⇓ |  |
|  | *SLP* |  |  |  | ⇑ |  |  |  |  |  | ⇑ |  |  |
|  |  |  |  |  |  |  |  |  |  |  |  |  |  |
| **7 am** | *T* |  |  |  | ⇑ | ⇓ |  |  |  |  | ⇓ |  |  |
|  | *T_w_* |  |  |  | ⇑ | ⇓ |  |  |  |  | ⇓ |  |  |
|  | *T_d_* |  |  |  |  | ⇓ |  |  |  |  | ⇓ |  |  |
|  | *RH* |  |  |  | ⇓ | ⇓ |  |  |  |  |  |  |  |
|  | *V* |  |  |  | ⇓ | ⇓ |  |  |  |  |  | ⇓ |  |
|  | *SLP* |  |  |  |  |  |  |  |  |  | ⇑ |  |  |
|  |  |  |  |  |  |  |  |  |  |  |  |  |  |
| **1 pm** | *T* |  | ⇑ |  |  | ⇓ |  |  |  |  | ⇑ | ⇑ |  |
|  | *T_w_* |  | ⇑ |  |  |  |  |  |  |  |  | ⇑ |  |
|  | *T_d_* |  |  |  |  | ⇓ |  |  |  |  |  |  |  |
|  | *RH* |  |  | ⇓ | ⇓ | ⇓ |  |  |  |  | ⇓ | ⇓ |  |
|  | *V* | ⇑ |  |  | ⇓ |  |  |  |  |  |  |  |  |
|  | *SLP* |  |  |  |  |  |  |  |  |  |  |  |  |
|  |  |  |  |  |  |  |  |  |  |  |  |  |  |
| **7 pm** | *T* |  | ⇑ |  | ⇑ | ⇑ |  |  |  |  |  | ⇑ |  |
|  | *T_w_* |  | ⇑ |  | ⇑ |  |  |  |  |  |  |  |  |
|  | *T_d_* |  |  |  |  | ⇓ |  |  |  |  |  |  |  |
|  | *RH* |  |  |  | ⇓ | ⇓ |  |  |  |  |  |  |  |
|  | *V* |  |  | ⇑ |  |  |  |  |  |  |  |  |  |
|  | *SLP* |  |  |  |  |  |  |  |  |  |  |  |  |
|  |  |  |  |  |  |  |  |  |  |  |  |  |  |
|  |  |  |  |  |  |  |  |  |  |  |  |  |  |

| **ORF** |  | **Jan** | **Feb** | **Mar** | **Apr** |  | **PHF** | **Mar** | **Apr** |  | **RIC** | **Feb** | **Mar** | **Apr** | **May** |
| --- | --- | --- | --- | --- | --- | --- | --- | --- | --- | --- | --- | --- | --- | --- | --- |
| **1 am** | *T* |  |  |  |  |  | **1 am** |  |  |  | **1 am** | ⇑ |  |  |  |
|  | *T_w_* |  |  |  |  |  |  |  |  |  |  | ⇑ |  |  |  |
|  | *T_d_* |  |  |  |  |  |  |  |  |  |  |  |  |  | ⇓ |
|  | *RH* |  |  |  |  |  |  |  |  |  |  |  |  | ⇓ | ⇓ |
|  | *V* |  |  |  |  |  |  |  |  |  |  |  |  |  |  |
|  | *SLP* |  |  |  |  |  |  |  |  |  |  |  |  |  |  |
|  |  |  |  |  |  |  |  |  |  |  |  |  |  |  |  |
| **7 am** | *T* |  |  |  |  |  | **7 am** |  |  |  | **7 am** | ⇑ |  |  |  |
|  | *T_w_* |  |  |  |  |  |  |  |  |  |  | ⇑ |  |  |  |
|  | *T_d_* |  |  |  |  |  |  |  |  |  |  |  |  |  | ⇓ |
|  | *RH* |  |  |  |  |  |  |  |  |  |  |  |  |  |  |
|  | *V* |  |  |  |  |  |  |  |  |  |  |  |  | ⇓ |  |
|  | *SLP* |  |  |  |  |  |  |  |  |  |  |  |  |  |  |
|  |  |  |  |  |  |  |  |  |  |  |  |  |  |  |  |
| **1 pm** | *T* |  | ⇑ | ⇑ |  |  | **1 pm** | ⇑ | ⇑ |  | **1 pm** | ⇑ |  |  | ⇑ |
|  | *T_w_* |  | ⇑ | ⇑ |  |  |  | ⇓ |  |  |  | ⇑ |  | ⇑ |  |
|  | *T_d_* |  |  |  |  |  |  |  |  |  |  | ⇑ |  |  |  |
|  | *RH* |  | ⇓ | ⇓ |  |  |  |  | ⇓ |  |  |  | ⇓ |  | ⇓ |
|  | *V* |  |  |  |  |  |  |  |  |  |  |  |  |  |  |
|  | *SLP* |  |  |  |  |  |  |  |  |  |  |  |  | ⇑ |  |
|  |  |  |  |  |  |  |  |  |  |  |  |  |  |  |  |
| **7 pm** | *T* |  | ⇑ | ⇑ | ⇑ |  | **7 pm** |  | ⇑ |  | **7 pm** | ⇑ |  | ⇑ |  |
|  | *T_w_* |  | ⇑ | ⇑ | ⇑ |  |  |  | ⇑ |  |  | ⇑ |  | ⇑ |  |
|  | *T_d_* |  | ⇑ | ⇑ |  |  |  |  |  |  |  | ⇑ |  |  |  |
|  | *RH* | ⇓ |  | ⇓ | ⇓ |  |  |  | ⇓ |  |  |  |  |  |  |
|  | *V* |  |  |  |  |  |  |  |  |  |  |  | ⇑ |  |  |
|  | *SLP* |  |  |  |  |  |  |  |  |  |  |  |  | ⇑ |  |
|  |  |  |  |  |  |  |  |  |  |  |  |  |  |  |  |
|  |  |  |  |  |  |  |  |  |  |  |  |  |  |  |  |

| **ROA** |  | **Jan** | **Feb** | **Mar** | **Apr** | **May** | **Jun** | **Jul** | **Aug** | **Sep** | **Oct** | **Nov** | **Dec** |
| --- | --- | --- | --- | --- | --- | --- | --- | --- | --- | --- | --- | --- | --- |
| **1 am** | *T* |  | ⇑ | ⇑ | ⇑ |  | ⇓ |  |  | ⇓ | ⇓ |  | ⇑ |
|  | *T_w_* |  | ⇑ | ⇑ |  | ⇓ | ⇓ | ⇓ | ⇓ | ⇓ | ⇓ |  | ⇑ |
|  | *T_d_* |  | ⇑ |  |  | ⇓ | ⇓ | ⇓ | ⇓ | ⇓ | ⇓ |  | ⇑ |
|  | *RH* |  | ⇓ | ⇓ | ⇓ | ⇓ | ⇓ | ⇓ | ⇓ | ⇓ |  |  |  |
|  | *V* |  |  | ⇓ | ⇓ | ⇓ | ⇓ | ⇓ |  | ⇓ | ⇓ | ⇓ | ⇓ |
|  | *SLP* | ⇑ | ⇑ | ⇑ | ⇑ | ⇑ | ⇑ | ⇑ |  | ⇑ | ⇑ | ⇑ | ⇑ |
|  |  |  |  |  |  |  |  |  |  |  |  |  |  |
| **7 am** | *T* |  | ⇑ | ⇑ |  | ⇓ | ⇓ |  | ⇓ | ⇓ | ⇓ |  | ⇑ |
|  | *T_w_* |  | ⇑ |  |  | ⇓ | ⇓ | ⇓ | ⇓ | ⇓ | ⇓ |  | ⇑ |
|  | *T_d_* |  | ⇑ |  |  | ⇓ | ⇓ | ⇓ | ⇓ | ⇓ | ⇓ |  | ⇑ |
|  | *RH* |  |  |  | ⇓ | ⇓ | ⇓ | ⇓ | ⇓ | ⇓ |  |  |  |
|  | *V* |  |  | ⇓ | ⇓ | ⇓ | ⇓ | ⇓ |  | ⇓ | ⇓ | ⇓ |  |
|  | *SLP* | ⇑ |  | ⇑ | ⇑ | ⇑ | ⇑ | ⇑ |  | ⇑ | ⇑ | ⇑ | ⇑ |
|  |  |  |  |  |  |  |  |  |  |  |  |  |  |
| **1 pm** | *T* | ⇑ | ⇑ | ⇑ | ⇑ | ⇑ | ⇑ | ⇑ | ⇑ | ⇑ | ⇑ | ⇑ | ⇑ |
|  | *T_w_* | ⇑ | ⇑ | ⇑ | ⇑ |  |  | ⇓ |  |  | ⇑ | ⇑ | ⇑ |
|  | *T_d_* |  |  | ⇑ |  | ⇓ | ⇓ | ⇓ | ⇓ | ⇓ |  |  | ⇑ |
|  | *RH* | ⇓ | ⇓ | ⇓ | ⇓ | ⇓ | ⇓ | ⇓ | ⇓ | ⇓ | ⇓ | ⇓ | ⇓ |
|  | *V* |  |  |  | ⇓ |  |  |  |  |  | ⇓ |  |  |
|  | *SLP* | ⇑ |  |  | ⇑ | ⇑ | ⇑ |  |  |  | ⇑ |  | ⇑ |
|  |  |  |  |  |  |  |  |  |  |  |  |  |  |
| **7 pm** | *T* | ⇑ | ⇑ | ⇑ | ⇑ | ⇑ | ⇑ | ⇑ | ⇑ | ⇑ | ⇑ | ⇑ | ⇑ |
|  | *T_w_* | ⇑ | ⇑ | ⇑ | ⇑ | ⇑ |  |  |  | ⇑ | ⇑ | ⇑ | ⇑ |
|  | *T_d_* |  | ⇑ | ⇑ |  | ⇓ | ⇓ | ⇓ | ⇓ |  |  | ⇑ | ⇑ |
|  | *RH* | ⇓ | ⇓ | ⇓ | ⇓ | ⇓ | ⇓ | ⇓ | ⇓ | ⇓ | ⇓ | ⇓ | ⇓ |
|  | *V* |  |  |  |  | ⇑ |  |  |  | ⇓ | ⇓ | ⇓ |  |
|  | *SLP* | ⇑ |  |  | ⇑ | ⇑ | ⇑ |  |  | ⇑ | ⇑ |  | ⇑ |
|  |  |  |  |  |  |  |  |  |  |  |  |  |  |
|  |  |  |  |  |  |  |  |  |  |  |  |  |  |

Supplemental Figure 1. Relative risk of mortality as a function of lag at specific high DTR values for each city (see Table 2). Error bars show 95% confidence intervals.

Supplemental Figure 2. Relative risk of mortality as a function of DTR and lag from a consensus model at each location.
